# Effect of a Third COVID-19 Vaccine Dose on the Incidence of Long COVID Among Adults Who Completed a Primary Vaccine Series: a Target Trial Emulation in a Community-Based Cohort

**DOI:** 10.1101/2025.11.03.25339444

**Published:** 2025-11-06

**Authors:** Yanhan Shen, Zach Shahn, McKaylee M. Robertson, Kelly Gebo, Denis Nash

**Affiliations:** 1. Institute for Implementation Science in Population Health (ISPH), City University of New York (CUNY); New York, NY, USA; 2. Department of Epidemiology and Biostatistics, Graduate School of Public Health and Health Policy, City University of New York (CUNY); New York, NY, USA; 3. Department of Medicine, Johns Hopkins University; Baltimore, MD, USA

**Keywords:** long COVID, COVID-19 vaccine, longitudinal, Sequential Target Trial Emulation, Intention to Treat, Per Protocol

## Abstract

**Background:**

Evidence on whether a third COVID-19 vaccine dose lowers long COVID risk is mixed. We estimated the effect of receiving ≥1 third dose versus completing only a primary series on 6- and 12-month long COVID incidence using a target-trial emulation in a U.S. community cohort.

**Methods:**

We analyzed the CHASING COVID Cohort, a prospective, community-based study of U.S. adults. Eligible participants were ≥18 years, had completed a two-dose primary series, had no prior long COVID, and had no SARS-CoV-2 infection in the 3 months before time zero. Strategies compared were: receive a third dose at time zero vs. not receive a third dose during follow-up. Long COVID was defined as ≥1 new symptom at or beyond 3 months post-infection with concurrent activity limitation, both absent in the prior year. Follow-up was 6 and 12 months. We used a per-protocol analog: participants were artificially censored upon deviating from their assigned strategy or lost-to-follow-up, with inverse-probability weights to address selection due to censoring and time-varying confounding. We fit weighted pooled logistic models to estimate weighted incidence, differences, and ratios at each horizon.

**Results:**

Across 16 sequential trials (18,930 person-trials; 4,044 unique individuals), 3,321 person-trials received a third dose at time zero and 15,609 did not. At 6 months, weighted long COVID incidence was 0.9% (95% CI, 0.5%, 1.3%) with a third dose vs. 1.0% (0.8%, 1.1%) without (risk difference (RD), −0.1%; 95% CI, −0.5%, 0.4%; risk ratio (RR), 0.93; 95% CI, 0.54, 1.44). At 12 months, incidence was 4.9% (4.1%, 5.9%) with a third dose vs. 4.5% (4.1%, 4.8%) without (RD, 0.4%; 95% CI, −0.5%, 1.4%; RR, 1.09; 95% CI, 0.90, 1.33).

**Conclusion:**

In this community-based target-trial emulation, receiving a third COVID-19 vaccine dose did not meaningfully reduce 6- or 12-month long COVID incidence compared with completing only a primary series.

## INTRODUCTION

A portion of individuals continue to experience symptoms three months following an acute COVID-19 infection that they had not experienced prior to infection, a condition collectively termed long COVID, which the National Academies describe as an infection-associated chronic state with persistent, relapsing, or progressive symptoms that often lead to activity limitations—such as impairments in daily home functioning, employment, or education^1–3^. Based on a nationally representative sample of U.S. adults as of December 2023, 8.4% (95% CI, 8.0%−8.8%) of adults reported they had ever experienced long COVID, 3.6% (95% CI, 3.3%−3.9%) reported they currently had long COVID, and 2.3% (95% CI, 2.1%−2.5%) reported they currently had activity-limiting long COVID, regardless of their COVID-19 infection history^4^.

Staying up to date on COVID-19 vaccines could prevent or reduce the burden of long COVID, either by preventing infection^5–10^ or by preventing or mitigating long COVID symptoms, given an infection^11,12^. The findings across studies have been mixed. Several studies found that completion of the third dose after completing the primary series was associated with diminished risk for long COVID, when compared to receipt of primary series alone or no vaccination at all^13–19^. In a Spanish cohort study, Domènech-Montoliu et al. found lower long COVID incidence after a third vaccine dose^13^. Two U.S. cross-sectional studies (Romeiser et al. and Xie et al.) similarly reported reduced odds of long COVID among those who received a third dose compared to partially vaccinated or unvaccinated individuals^14,15^. In contrast, other studies have found no clear protective effect of booster doses^20,21^. Ballouz et al. conducted a pooled analysis of two prospective longitudinal cohorts, found that booster vaccination did not consistently reduce the incidence of long COVID across SARS-CoV-2 variants^20^. In a cross-sectional study, Kahlert et al. similarly found no significant difference between those who received booster doses to those who had received only the primary series or remained unvaccinated^21^. These mixed findings underscore ongoing uncertainty about whether COVID-19 vaccination beyond the primary series reduces the risk of long COVID.

The reasons for mixed results on the role of COVID vaccines in preventing or reducing the burden of long COVID are likely multifactorial, including difference in study design, population characteristics, evolving immunity, circulating variants, availability of treatments (e.g., monoclonal antibodies, Paxlovid), and varying definitions of long COVID across studies. First, many studies employed cross-sectional designs, which are prone to selection biases, as individuals with persistent symptoms might be more motivated to participate, potentially inflating prevalence estimates. In addition, because data were captured at a single point in time, these studies were unable to assess incidence, limiting the feasibility of causal inference. Second, most existing studies have relied on electronic health records (EHR) or claims data, which are limited to individuals who have accessed healthcare and may not capture the broader community, thereby limiting generalizability. Third, relatively few studies adopted target trial emulation (TTE) designs, which are a recommended approach for strengthening causal inference in observational studies and mitigate biases like immortal time bias. These gaps underscore the need for methodologically rigorous studies of community-based populations to establish clearer causal relationships. To address this, we used a community-based prospective cohort not restricted to healthcare system enrollees and applied a TTE framework to estimate the impact of receiving at least a third (v.s. no third dose) COVID-19 vaccine on the incidence of 6- and 12-month long COVID among participants who completed a two-dose primary series vaccine.

## METHODS

### Participants

The Communities, Households, and SARS-CoV-2 Epidemiology (CHASING) COVID Cohort study, launched on March 28, 2020, was a national, community-based prospective cohort study of adults residing in the U.S. or its territories^22^. Through internet-based recruitment methods, the cohort represents a socio-demographically and geographically diverse sample of U.S. adults. Recruitment and follow-up procedures have been detailed in previous publications^23–25^. Between March 2020 and December 2023, participants completed approximately quarterly online assessments covering a range of topics, including health insurance coverage, food insecurity, housing instability, SARS-CoV-2 infection, long COVID symptoms, activity limitations, and COVID-19 vaccination receipt. To detect infection-induced seroconversion, dried blood spot (DBS) samples were collected and tested in four rounds between 2020 and 2023 for total antibodies to the SARS-CoV-2 nucleocapsid protein using the Bio-Rad Platelia assay, which detects IgA, IgM, and IgG, with manufacturer-reported sensitivity of 98.0% and specificity of 99.3%^26–28^. The study was approved by the Institutional Review Board of the City University of New York (CUNY).

### Exposure: the Third Dose of COVID-19 Vaccine

Participants reported detailed information on their COVID-19 vaccinations across 13 follow-up assessments conducted between December 2020 and December 2023, including the number of doses received, vaccination dates for each dose, and the vaccine manufacturer. For this analysis, we focused on individuals who completed both doses of two-dose primary vaccine series. This included vaccines approved to be administered in the U.S.: Pfizer-BioNTech, Moderna, and Novavax^29–31^. Participants who received the Johnson & Johnson (J&J) vaccine were excluded, as only about 5% of participants received J&J and its single-dose mechanism complicates interpretation of third-dose vaccination following a two-dose primary series, which was the focus of this analysis. The date of primary series completion was defined as the date of receipt of the second dose. The exposure of interest in this study was the receipt of a third COVID-19 vaccine dose. This third dose included either the 2021–2022 monovalent booster or the 2022–2023 bivalent booster, administered after the primary series^32,33^.

### Outcome: Long COVID Status

The primary long COVID case definition was based on case definitions from the World Health Organization (WHO)^34^, the Centers for Disease Control and Prevention (CDC)^1,35^, and the National Academies of Science, Engineering, and Medicine (NASEM)^2^. We defined individuals with long COVID as participants who experienced at least one symptom and concurrent activity limitations between 3 and 12 months post-infection, neither of which was present in the year preceding infection. The long COVID symptoms include fatigue, post-exertional malaise, trouble concentrating/brain fog, dizziness, erratic heartbeat, gastrointestinal issues, and loss or alteration of taste or smell. These symptoms were assessed roughly every three months between November 2020 and December 2023. Activity limitations were assessed concurrently with long COVID symptoms and defined as difficulty performing daily activities or household responsibilities^36^. The details of infection and infection date assignment was documented in Appendix 1.

### Study design: Sequential Target Trial Emulation Using a Cohort

This study aimed to estimate the effect of receiving at least a third COVID-19 vaccine dose on the 6- and 12-month incidence of long COVID compared to only completing a two-dose primary vaccine series. We employed TTE, a framework for designing and analyzing observational studies to address causal questions when a randomized controlled trial (RCT) is impractical due to ethical, logistical, or resource constraints. The TTE approach attempts to estimate the same effect that would be estimated by a hypotehtical RCT by explicitly specifying elements of the hypothetical RCT, including clear definitions of eligibility criteria, treatment strategies, the alignment of time zero (start of follow-up), and outcomes assessment. We first specified the hypothetical RCT protocol, then implemented the observational emulation of this protocol^37^. Furthermore, our nested sequential trial design maximizes statistical efficiency and enhances precision by utilizing available longitudinal data. Detailed specifications of the emulated sequential target trial were provided in Supplementary Table 1.

#### Eligibility Criteria and Treatment Strategy

Participants were eligible for inclusion if they were aged 18 years or older and had received both doses of a two-dose primary COVID-19 vaccine series (Pfizer-BioNTech, Moderna, or Novavax). Participants were excluded if they had any prior history of long COVID (as defined above), a documented SARS-CoV-2 infection within the three months preceding the start of follow-up (time zero), or had received any additional COVID-19 vaccine doses beyond the two-dose primary series before that trial’s time zero (Supplementary Figure 1). Participants were assigned to one of two treatment strategies: receiving at least a third dose of COVID-19 vaccine or not receiving a third dose.

#### Sequential Trials, Time Zero, and Follow Up

To account for and leverage participants meeting eligibility criteria at different time points, a series of sequential target trials were emulated monthly over a 16-month period, from September 2021 (when third doses first became publicly available in the U.S.) through December 2022^38^. Time zero for each sequential trial was defined as follows: For exposed individuals, time zero was the date they received their third dose of the COVID-19 vaccine. In the same month, unexposed individuals—those who had not received a third dose by the end of the month—were identified, and the last day of that month was assigned as their time zero. Eligibility for each sequential trial was determined based on time-updated, participant-specific characteristics. The sequential trial design is illustrated in Supplementary Figure 1&2.

The primary outcomes were the 6- and 12-month incidence of long COVID, assessed from trial time zero. Participants were followed until the earliest occurrence of one of the following events: meeting criteria for long COVID, loss to follow-up (defined as any missed follow-up assessment), or the end of the designated follow-up period (6 and 12 months from time zero). Once participants were censored, they did not re-enter the sample for that trial.

#### Per-protocol Effects

Randomization of the two exposure groups was emulated by adjusting for confounders of the receipt of the third COVID-19 vaccine dose at time zero and subsequent incidence of long COVID. To further account for evolving SARS-CoV-2 variants and changes in state government guidelines regarding booster dose eligibility, calendar month at time zero was also included and modeled using a natural cubic spline with 4 degrees of freedom to flexibly capture nonlinear time trends.

We assessed the per-protocol (PP) effects as our primary causal contrast of interest. This effect represents the difference in the expected incidence of long COVID under two hypothetical scenarios: one in which all participants had received at least a third dose of the COVID-19 vaccine at time zero, and another in which no participants had received a third dose at any point during follow-up. In this framework, receipt of additional vaccine doses beyond the third was not considered a deviation from the treatment strategy.

### Statistical Analysis

#### Time Zero Characteristics and Covariate Balance

Descriptive statistics for time zero characteristics were presented as mean with standard deviation (SD), median with interquartile range (IQR), or frequencies with proportions (details of these characteristics were provided in Supplementary Files Appendix 2). Standardized mean differences (SMDs) were used to compare characteristics of unique participants who received at least a third dose of COVID-19 vaccine as of December 2022 verse those who only received two doses of primary vaccine, as well as person-trial comparisons between those who received a third dose verse those who did not at time zero. Additionally, weighted SMDs were calculated using inverse probability of treatment weighting (IPTW) combined with inverse probability of censoring weighting (IPCW) under the per-protocol framework to assess covariates balance in the analytic sample. An SMD of 0.1 or greater is often interpreted as indicating a meaningful imbalance in covariates as a rule of thumb^39–41^.

#### Estimating Per-Protocol (PP) Effects

We employed an IPTW approach to adjust for time-fixed and time-varying confounding (details of these characteristics were provided in Supplementary Files Appendix 2)^42,43^. We used IPCW to adjust for potential selection bias introduced by artificial censoring. We assumed that factors associated with loss to follow-up or deviation from the treatment arm at the time of censoring included: age, gender, race/ethnicity, education, household income, comorbidities, prior SARS-CoV-2 infection history, obesity status, smoking status, health insurance status, access to a primary care provider, region of residence, food insecurity, housing instability, and calendar month of censoring. Spline terms for age and calendar month were also included in the IPCW models to flexibly capture nonlinear relationships. We estimated the time-varying weights by fitting a pooled logistic model for the monthly probability of remaining uncensored, including variables covariates described above.

The PP analysis, person-time was censored at the earliest occurrence of either deviation from the assigned treatment strategy—defined as receiving a third dose in the control group —or loss to follow-up during the 6- and 12-month follow-up periods. To estimate the effect of a third dose of COVID-19 vaccine on risk of long COVID, we fitted a pooled logistic regression model weighted by the product of IPTW and IPCW. The model included treatment arm and follow-up time, with additional interaction terms between treatment and both linear and quadratic time to allow for time-varying effects. We used the predicted probabilities from this weighted model to estimate discrete hazards and calculate adjusted cumulative incidence under each treatment strategy. Risks were derived from survival probabilities, computed as the cumulative product of one minus the estimated hazard at each time point. From these estimates, we calculated the 6- and 12-month cumulative incidence (risk), risk differences, and risk ratios. Percentile-based 95% confidence intervals (CIs) were obtained using nonparametric bootstrapping with 500 samples. Weighted cumulative incidence curves for each treatment group were also estimated using the same model to visualize changes in risk over time^44^.

#### Sensitivity Analysis

We conducted the following sensitivity analyses to assess the robustness of our findings. (1) To explore variations in long COVID case definitions, we conducted sensitivity analyses using three alternative definitions (Supplementary Table 2A&B). The first alternative definition required at least one symptom at least 3 months post-infection, which was absent pre-infection. The second alternative definition required at least one symptom to be present at two distinct time points: an initial occurrence 3 months post-infection, followed by a second occurrence at least 60 days later. The symptoms at these two time points were not required to be the same, but both had to be absent in the year preceding the index infection date. The third alternative definition classified cases based on self-identified long COVID, defined as “experiencing symptoms more than 4 weeks after you first had COVID-19 that are not explained by something else?”, based on the U.K.’s Office for National Statistics (ONS) case definition that was collected in each follow-up assessment since June 2022^45,46^. These alternative definitions allowed us to assess the sensitivity of our findings under different criteria for long COVID classification. (2) We conducted the sensitivity analysis separately for individuals who were infection naïve to SARS-CoV-2 as of time zero, to account for potential differences in hybrid immunity, prior exposure, and risk of long COVID (Supplementary Table 3). We used SAS version 9.4 (SAS Institute, Cary, NC, USA) for data management and R version 2024.09.0+375 (R Foundation for Statistical Computing, Vienna, Austria) for data analyses.

## RESULTS

A total of 18,930 person-trials were emulated across 16 sequential target trials, with 3,321 person-trials in which participants receive a third COVID-19 vaccine dose at time zero and 15,609 person-trials in which participants did not (Table 1). Before weighting, participants who received a third COVID-19 vaccine dose were more likely to be older, with 12.1% aged 65 or older compared to 8.3% in the group did not receive a third dose (SMD=0.191). In terms of educational attainment, 71.9% of individuals who received a third COVID-19 vaccine dose had completed college or higher education compared to 56.5% of participants who did not receive a third dose (SMD=0.326). Additionally, individuals who received a third COVID-19 vaccine dose were less likely to report food insecurity (15.7% vs. 27.7%, SMD=0.297) compared to their counterparts who did not receive a third dose. After IPTW, these imbalances were markedly reduced: the weighted SMDs for age ≥ 65, education, and food insecurity fell to 0.013, 0.089, and 0.088, respectively.

In the per-protocol (PP) analysis, the 6-month weighted incidence of long COVID, as defined by at least one symptom persisting for three months post-infection and concurrent activity limitation which were absent before infection, was 0.9% (95% CI: 0.5%, 1.3%) in the group who received a third COVID-19 vaccine dose and 1.0% (95% CI: 0.8%, 1.1%) in the group who did not receive a third dose, with an incidence difference of −0.1% (95% CI: −0.5%, 0.4%) and an incidence ratio of 0.929 (95% CI: 0.544, 1.437) (Table 2). A similar pattern was observed for other case definitions, such as symptoms persisting for three months at two time points, where the standardized incidence was 1.2% (95% CI: 1.0%, 1.4%) in the group who received a third COVID-19 vaccine dose and 1.3% (95% CI: 0.8%, 1.8%) in the group who did not receive a third dose, yielding a incidence difference of 0% (95% CI: −0.4%, 0.6%) and a incidence ratio of 1.032 (95% CI: 0.659, 1.577) (Supplementary Table 2A).

**Figure 1:**
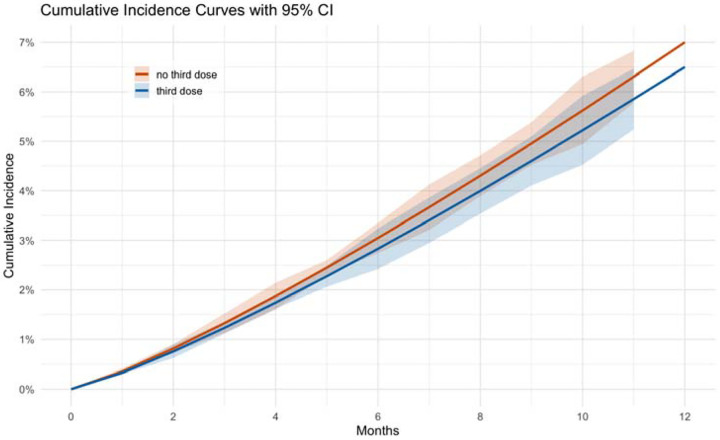
Estimated cumulative incidence of long COVID curves by the third dose vaccination status across 16 monthly sequential target trials between September 2021 and December 2023

For the 12-month PP analysis, individuals who received a third COVID-19 vaccine dose had an incidence of 4.9% (95% CI: 4.1%, 5.9%) compared to 4.5% (95% CI: 4.1%, 4.8%) in the group who did not receive a third dose, with an incidence difference of 0.4% (95% CI: −0.5%, 1.4%) and an incidence ratio of 1.092 (95% CI: 0.897, 1.329) (Table 2). For other definitions, such as symptoms persisting twice at three months, the incidence difference was 0.9% (95% CI: −0.1%, 2.1%) and the incidence ratio was 1.184 (95% CI: 0.989, 1.390) (Supplementary Table 2B). Cumulative incidence curves showed overlapping trends for the groups who received a third COVID-19 vaccine dose and the group who did not receive a third dose (Figure 1).

## DISCUSSION

This study investigated the relationship between receiving a third dose of the COVID-19 vaccine (booster dose) and the 6- and 12-month incidence of long COVID among a U.S. community-based cohort. Our results showed no meaningful differences in the incidences of long COVID between the group who received at least a third COVID-19 vaccine dose and the group who did not in sequential prospective target trials. Notably, the null studies and our analysis tend to share designs that capture milder or community-reported infections and rely on participant-reported outcomes, whereas studies reporting a protective effect more often used electronic health records or claims data, which may preferentially sample individuals with more severe illness or healthcare contact. Thus, differences in case-ascertainment—particularly whether individuals with milder long COVID are included—may partly explain why some investigations observe an effect and others do not.

These findings contrast with several studies that suggested booster vaccination reduces risk of long COVID. The discrepancies can be attributed to several factors. Firstly, differences in study design, such as the cross-sectional approaches used by Domènech-Montoliu et al. and Romeiser et al. compared to our target trial emulation within a prospective community-based cohort, may explain the inconsistencies^13,14^. Secondly, variability in long COVID definitions, ranging from persistent symptoms (Xie et al.) to broader measures including activity limitations (Di Fusco et al.), further complicates comparisons^15,16^. We addressed this by testing multiple case definitions in sensitivity analyses, including alternative measures of symptom persistence and functional impact. Thirdly, behavioral factors, such as healthcare utilization disparities noted by Nguyen et al. and differences in vaccine types and schedules, as highlighted by Wee et al., may also contribute^18,19^. These complexities emphasize the need for harmonized definitions and methodologies in future research.

A key strength of this study is the use of TTE design, which enhances causal inference by aligning observational design with the framework of RCT. Unlike standard approaches, TTE explicitly defines treatment strategies and time zero in advance, ensuring that eligibility, exposure classification, and follow-up begin simultaneously^47^. This structure helps prevent immortal time bias—a form of bias that arises when participants must survive a certain period to receive the exposure, thus artificially lowering event rates in the exposed group^48^. By emulating a sequence of trials over time, our design not only mitigate this bias but also improves statistical efficiency by incorporating all eligible person-time.

Despite its strengths, this study has limitations. First, long COVID outcomes relied on self-reported symptoms and conditions, which may introduce recall and reporting biases. Second, our sample excluded individuals with prior SARS-CoV-2 infections within three months of time zero, potentially limiting generalizability to populations with differing exposure histories. However, this approach aligns with CDC guidance, which suggests delaying COVID-19 vaccination for up to three months after infection, as recent infection offers some immunity^49^. This strategy also helps reduce immortal time bias by excluding individuals who may have been temporarily at lower risk of reinfection during that period. Third, although our TTE design adjusted for a comprehensive set of confounders, unmeasured factors, such as health literacy differences between staying up-to-date vaccinated individuals and vaccinated once individuals, may still confound the results. Lastly, infection imputation algorithms—while designed to enhance detection of infections using repeated serologic and self-report data, may still be susceptible to misclassification. To reduce this risk, we applied conservative criteria when assigning infection dates; for instance, serology-identified infections were only assigned dates when supported by viral test results or Council of State and Territorial Epidemiologists criteria that helped narrow the plausible infection window. Nonetheless, some residual misclassification may persist due to limitations in accurately pinpointing the true infection date. Therefore, these limitations should be considered when interpreting the study’s findings.

Our findings suggested that receiving at least a third dose of the COVID-19 vaccine may not substantially reduce the risk of long COVID compared to only receiving the primary vaccine in a community-based cohort. These results underscored the need for further research employing rigorous analytical methods to clarify the relationship between vaccination and long COVID. Future studies could potentially benefit from prospective designs that include physician-diagnosed long COVID outcomes to reduce the risk of outcome misclassification and better collect potential differences (e.g. healthcare-seeking behavior and health literacy differences) between individuals who are up-to-date on COVID-19 vaccination and those who received only the primary vaccines.

## Supplementary Material

Supplement 1

## Figures and Tables

**Table 1:** Time-zero characteristics of eligible participants among person-trial population by COVID-19 vaccine status across 16 sequential trial, The CHASING COVID Cohort Study, September 2021 – December 2023

	Received the third dose	Not received the third dose	SMD*	Weighted SMD
	N=3,321	N=15,609		
**Age category**				
18–29	657 (19.8%)	3883 (24.9%)	0.191	0.013
30–39	891 (26.8%)	4590 (29.4%)		
40–49	603 (18.2%)	2878 (18.4%)		
50–64	769 (23.2%)	2956 (18.9%)		
>=65	401 (12.1%)	1302 (8.3%)		
**Gender**				
Male	1538 (46.3%)	6926 (44.4%)	0.041	0.026
Female	1677 (50.5%)	8205 (52.6%)		
Non-Binary	106 (3.2%)	478 (3.1%)		
**Race/Ethnicity**				
Hispanic	462 (13.9%)	2946 (18.9%)	0.256	0.072
White Non-Hispanic	2247 (67.7%)	9053 (58.0%)		
Black Non-Hispanic	236 (7.1%)	2005 (12.8%)		
Asian Non-Hispanic	290 (8.7%)	1180 (7.6%)		
Others	86 (2.6%)	425 (2.7%)		
**Education**				
No College	932 (28.1%)	6786 (43.5%)	0.326	0.089
College and above	2389 (71.9%)	8823 (56.5%)		
**Household annual income**				
<35k or unknown	788 (23.7%)	5111 (32.7%)	0.248	0.073
35–100k	1420 (42.8%)	6756 (43.3%)		
>100k	1113 (33.5%)	3742 (24.0%)		
**Health Insurance Status**				
No	223 (6.7%)	2024 (13.0%)	0.187	0.075
Yes	3098 (93.3%)	13585 (87.0%)		
**Food insecure**				
No	2801 (84.3%)	11286 (72.3%)	0.297	0.088
Yes	520 (15.7%)	4323 (27.7%)		
**Housing Instable**				
No	3028 (91.2%)	13038 (83.5%)	0.233	0.047
Yes	293 (8.8%)	2571 (16.5%)		
**Region**				
MidWest	590 (17.8%)	2664 (17.1%)	0.182	0.045
North East	**1104 (33.2%)**	**4257 (27.3%)**		
South	764 (23.0%)	4741 (30.4%)		
West	832 (25.1%)	3767 (24.1%)		
US Terr or Unknown	31 (1.0%)	180 (1.2%)		
**Access to primary care doctor**				
No	744 (22.4%)	4303 (27.6%)	0.158	0.102
Yes	2531 (76.2%)	10672 (68.4%)		
Don’t know	46 (1.4%)	634 (4.1%)		
**Current Smoker Status**				
No	2806 (84.5%)	11637 (74.6%)	0.248	0.087
Yes	515 (15.5%)	3972 (25.4%)		
**Obesity Status**				
No	2367 (71.3%)	10807 (69.2%)	0.045	0.003
Yes	954 (28.7%)	4802 (30.8%)		
**Cancer**				
No	3175 (95.6%)	15086 (96.6%)	0.054	0.059
Yes	146 (4.4%)	523 (3.4%)		
**Kidney disease**				
No	3267 (98.4%)	15371 (98.5%)	0.008	0.025
Yes	54 (1.6%)	238 (1.5%)		
**Lung disease**				
No	3245 (97.7%)	15140 (97.0%)	0.045	0.001
Yes	76 (2.3%)	469 (3.0%)		
**Type-2 diabetes**				
No	3086 (92.9%)	14445 (92.5%)	0.015	0.003
Yes	235 (7.1%)	1164 (7.5%)		
**Heart diseases**				
No	3241 (97.6%)	15144 (97.0%)	0.035	0.016
Yes	80 (2.4%)	465 (3.0%)		
**HIV/AIDS**				
No	3188 (96.0%)	14917 (95.6%)	0.021	0.025
Yes	133 (4.0%)	692 (4.4%)		
**Mental health disorders**				
No	2185 (65.8%)	9514 (61.0%)	0.101	0.048
Yes	1136 (34.2%)	6095 (39.0%)		
**Immunosuppression**				
No	3227 (97.2%)	15280 (97.9%)	0.047	0.064
Yes	94 (2.8%)	329 (2.1%)		

* SMD: standard mean difference

**Table 2:** Analog of the Per-Protocol IPTW and IPCW weighted incidence, incidence difference, and incidence ratio (95% confidence intervals) for 6- and 12-month long COVID, The CHASING COVID Cohort, September 2021 – December 2023

Follow up time	Treatment Strategy	Weighted risk (95% CI)		Weighted Risk difference (95% CI)		Weighted Risk ratio (95% CI)	
6 months	Participants who received a third COVID-19 vaccine dose	0.9%	0.5%, 1.3%	−0.1%	−0.5%, 0.4%	0.929	0.544, 1.437
	Participants who did not receive a third COVID-19 vaccine dose	1.0%	0.8%, 1.1%	ref	ref	ref	ref
12 months	Participants who received a third COVID-19 vaccine dose	4.9%	4.1%, 5.9%	0.4%	−0.5%, 1.4%	1.092	0.897, 1.329
	Participants who did not receive a third COVID-19 vaccine dose	4.5%	4.1%, 4.8%	ref	ref	Ref	ref

* Long COVID was defined as the occurrence of ≥1 new symptom and concurrent activity limitation between 3–12 months post-infection, both of which were absent in the year preceding infection.

## Data Availability

The data underlying this study contain potentially identifiable health information and are not publicly available due to ethical and legal restrictions. De-identified data may be shared on reasonable request and subject to institutional approvals and a Data Use Agreement. Requests will be reviewed case-by-case by the study investigators and the CUNY ISPH data governance/IRB. Please contact the corresponding author.

## References

[1] CDC. Long COVID basics. COVID-19. July 29, 2024. Accessed August 26, 2024. https://www.cdc.gov/covid/long-term-effects/index.html

[2] Ely EW, Brown LM, Fineberg HV, National Academies of Sciences, Engineering, and Medicine Committee on Examining the Working Definition for Long Covid. Long covid defined. N Engl J Med. Published online July 31, 2024. doi:10.1056/NEJMsb2408466

[3] National Academies of Sciences, Engineering, and Medicine; Health and Medicine Division; Board on Global Health; Board on Health Sciences Policy; Committee on Examining the Working Definition for Long COVID. A Long COVID Definition: A Chronic, Systemic Disease State with Profound Consequences. National Academies Press (US); 2024. 39110819

[4] Vahratian A, Saydah S, Bertolli J, Unger ER, Gregory CO. Prevalence of post-COVID-19 condition and activity-limiting post-COVID-19 condition among adults. JAMA Netw Open. 2024;7(12):e2451151. 39671200 10.1001/jamanetworkopen.2024.51151 PMC11645641

[5] Xu J, Lan X, Zhang L, The effectiveness of the first dose COVID-19 booster vs. full vaccination to prevent SARS-CoV-2 infection and severe COVID-19 clinical event: a meta-analysis and systematic review of longitudinal studies. Front Public Health. 2023;11:1165611. 37325336 10.3389/fpubh.2023.1165611 PMC10267329

[6] Chenchula S, Karunakaran P, Sharma S, Chavan M. Current evidence on efficacy of COVID-19 booster dose vaccination against the Omicron variant: A systematic review. J Med Virol. 2022;94(7):2969–2976. 35246846 10.1002/jmv.27697 PMC9088621

[7] Nealon J, Mefsin YM, McMenamin ME, Ainslie KEC, Cowling BJ. Reported effectiveness of COVID-19 monovalent booster vaccines and hybrid immunity against mild and severe Omicron disease in adults: A systematic review and meta-regression analysis. Vaccine X. 2024;17(100451):100451. 38379667 10.1016/j.jvacx.2024.100451 PMC10877401

[8] Song S, Madewell ZJ, Liu M, A systematic review and meta-analysis on the effectiveness of bivalent mRNA booster vaccines against Omicron variants. Vaccine. 2024;42(15):3389–3396. 38653679 10.1016/j.vaccine.2024.04.049

[9] Cheng MQ, Li R, Weng ZY, Song G. Relative effectiveness of bivalent COVID-19 vaccine: a systematic review and meta-analysis. Front Med (Lausanne). 2024;10:1322396. 38384317 10.3389/fmed.2023.1322396 PMC10879625

[10] Chenchula S, Chandra MB, Adusumilli MB, Immunogenicity, clinical efficacy and safety of additional second COVID-19 booster vaccines against Omicron and its subvariants: A systematic review. Rev Med Virol. 2024;34(1):e2515. 38282403 10.1002/rmv.2515

[11] Català M, Mercadé-Besora N, Kolde R, The effectiveness of COVID-19 vaccines to prevent long COVID symptoms: staggered cohort study of data from the UK, Spain, and Estonia. Lancet Respir Med. 2024;12(3):225–236. 38219763 10.1016/S2213-2600(23)00414-9

[12] Ceban F, Kulzhabayeva D, Rodrigues NB, COVID-19 vaccination for the prevention and treatment of long COVID: A systematic review and meta-analysis. Brain Behav Immun. 2023;111:211–229. 36990297 10.1016/j.bbi.2023.03.022 PMC10067136

[13] Domènech-Montoliu S, Puig-Barberà J, Badenes-Marques G, Long COVID prevalence and the impact of the third SARS-CoV-2 vaccine dose: A cross-sectional analysis from the third follow-up of the Borriana cohort, Valencia, Spain (2020–2022). Vaccines (Basel). 2023;11(10). doi:10.3390/vaccines11101590 PMC10611325 37896992

[14] Romeiser JL, Schoeneck K. COVID-19 booster vaccination status and long COVID in the United States: A nationally representative cross-sectional study. Vaccines (Basel). 2024;12(6):688. 38932418 10.3390/vaccines12060688 PMC11209278

[15] Xie Z, Stallings-Smith S, Patel S, Case S, Hong YR. COVID-19 booster vaccine uptake and reduced risks for long-COVID: A cross-sectional study of a U.S. adult population. Vaccine. 2024;42(16):3529–3535. 38670844 10.1016/j.vaccine.2024.04.070

[16] Di Fusco M, Sun X, Moran MM, Impact of COVID-19 and effects of booster vaccination with BNT162b2 on six-month long COVID symptoms, quality of life, work productivity and activity impairment during Omicron. J Patient Rep Outcomes. 2023;7(1):77. 37486567 10.1186/s41687-023-00616-5 PMC10366033

[17] Iba A, Hosozawa M, Hori M, Booster vaccination and post COVID-19 condition during the Omicron variant-dominant wave: A large population-based study. Clin Microbiol Infect. Published online December 9, 2024. doi:10.1016/j.cmi.2024.12.002 39662823

[18] Nguyen KH, McChesney C, Patel R, Bednarczyk RA, Vasudevan L, Corlin L. Association between COVID-19 booster vaccination and COVID-19 outcomes among U.s. adults. Vaccines (Basel). 2024;12(5):503. 38793754 10.3390/vaccines12050503 PMC11125699

[19] Wee LE, Lim JT, Goel M, Bivalent boosters and risk of post-acute sequelae following vaccine-breakthrough SARS-CoV-2 Omicron infection: a cohort study. Clin Infect Dis. Published online December 5, 2024. doi:10.1093/cid/ciae598 39657038

[20] Ballouz T, Menges D, Kaufmann M, Post COVID-19 condition after Wildtype, Delta, and Omicron SARS-CoV-2 infection and prior vaccination: Pooled analysis of two population-based cohorts. PLoS One. 2023;18(2):e0281429. 36812215 10.1371/journal.pone.0281429 PMC9946205

[21] Kahlert CR, Strahm C, Güsewell S, Post-acute sequelae after severe acute respiratory syndrome Coronavirus 2 infection by viral variant and vaccination status: A multicenter cross-sectional study. Clin Infect Dis. 2023;77(2):194–202. 36905145 10.1093/cid/ciad143 PMC10371307

[22] CHASING COVID cohort study. CUNY ISPH. Accessed September 6, 2024. https://cunyisph.org/chasing-covid/

[23] Robertson MM, Kulkarni SG, Rane M, Cohort profile: a national, community-based prospective cohort study of SARS-CoV-2 pandemic outcomes in the USA—the CHASING COVID Cohort study. BMJ Open. 2021;11(9):e048778. 10.1136/bmjopen-2021-048778 PMC8458000 34548354

[24] Nash D, Srivastava A, Shen Y, Seroincidence of SARS-CoV-2 infection prior to and during the rollout of vaccines in a community-based prospective cohort of U.S. adults. Sci Rep. 2024;14(1). doi:10.1038/s41598-023-51029-9 PMC10770061 38182731

[25] Nash D, Rane MS, Chang M, SARS-CoV-2 incidence and risk factors in a national, community-based prospective cohort of U.S. adults. medRxiv. Published online October 12, 2021. doi:10.1101/2021.02.12.21251659

[26] Tré-Hardy M, Wilmet A, Beukinga I, Analytical and clinical validation of an ELISA for specific SARS-CoV-2 IgG, IgA, and IgM antibodies. J Med Virol. 2021;93(2):803–811. 32667733 10.1002/jmv.26303 PMC7405491

[27] Trabaud MA, Icard V, Milon MP, Bal A, Lina B, Escuret V. Comparison of eight commercial, high-throughput, automated or ELISA assays detecting SARS-CoV-2 IgG or total antibody. J Clin Virol. 2020;132:104613. 32942137 10.1016/j.jcv.2020.104613 PMC7476603

[28] Plaga A, Wei R, Olson E, Evaluation of the clinical performance of 7 serological assays for SARS-CoV-2 for use in clinical laboratories. J Appl Lab Med. 2021;6(4):998–1004. 33825844 10.1093/jalm/jfab038 PMC8083591

[29] Office of the Commissioner. FDA approves first COVID-19 vaccine. U.S. Food and Drug Administration. August 9, 2024. Accessed October 11, 2024. https://www.fda.gov/news-events/press-announcements/fda-approves-first-covid-19-vaccine

[30] Moderna receives full U.s. fda approval for COVID-19 vaccine spikevax. Accessed January 4, 2025. https://investors.modernatx.com/news/news-details/2022/Moderna-Receives-Full-U.S.-FDA-Approval-for-COVID-19-Vaccine-Spikevax/default.aspx

[31] U.S. fda grants emergency use authorization for Novavax COVID-19 vaccine, Adjuvanted for individuals aged 18 and over. Accessed January 4, 2025. https://ir.novavax.com/press-releases/2022-07-13-U-S-FDA-Grants-Emergency-Use-Authorization-for-Novavax-COVID-19-Vaccine,-Adjuvanted-for-Individuals-Aged-18-and-Over

[32] Office of the Commissioner. Coronavirus (COVID-19) update: FDA takes additional actions on the use of a booster dose for COVID-19 vaccines. U.S. Food and Drug Administration. October 21, 2021. Accessed January 4, 2025. https://www.fda.gov/news-events/press-announcements/coronavirus-covid-19-update-fda-takes-additional-actions-use-booster-dose-covid-19-vaccines

[33] Rosenblum HG, Wallace M, Godfrey M, Interim recommendations from the Advisory Committee on Immunization Practices for the use of bivalent booster doses of COVID-19 vaccines - United States, October 2022. MMWR Morb Mortal Wkly Rep. 2022;71(45):1436–1441. 36355612 10.15585/mmwr.mm7145a2 PMC9707353

[34] Soriano JB, Murthy S, Marshall JC, Relan P, Diaz JV. WHO Clinical Case Definition Working Group on Post-COVID-19 Condition. A clinical case definition of post-COVID-19 condition by a Delphi consensus. Lancet Infect Dis. 2022;22(4):e102–e107. 34951953 10.1016/S1473-3099(21)00703-9 PMC8691845

[35] CDC. Clinical overview of Long COVID. COVID-19. August 14, 2024. Accessed August 26, 2024. https://www.cdc.gov/covid/hcp/clinical-overview/?CDC_AAref_Val=https://www.cdc.gov/coronavirus/2019-ncov/hcp/clinical-care/post-covid-conditions.html

[36] Stevens AC, Courtney-Long EA, Okoro CA, Carroll DD. Comparison of 2 disability measures, behavioral risk factor surveillance system, 2013. Prev Chronic Dis. 2016;13(160080). doi:10.5888/pcd13.160080 PMC4993115 27513997

[37] Hernán MA, Wang W, Leaf DE. Target trial emulation: A framework for causal inference from observational data. JAMA. 2022;328(24):2446–2447. 36508210 10.1001/jama.2022.21383

[38] Fast HE, Zell E, Murthy BP, Booster and additional primary dose COVID-19 vaccinations among adults aged ≥65 years - United States, August 13, 2021-November 19, 2021. MMWR Morb Mortal Wkly Rep. 2021;70(50):1735–1739. 34914672 10.15585/mmwr.mm7050e2 PMC8675661

[39] Hedges LV. Interpretation of the standardized mean difference effect size when distributions are not normal or homoscedastic. Educ Psychol Meas. Published online October 6, 2024:00131644241278928. 10.1177/00131644241278928 PMC11562970 39554777

[40] Austin PC. Balance diagnostics for comparing the distribution of baseline covariates between treatment groups in propensity - score matched samples. Stat Med. 2009;28(25):3083–3107. 19757444 10.1002/sim.3697 PMC3472075

[41] Normand ST, Landrum MB, Guadagnoli E, Validating recommendations for coronary angiography following acute myocardial infarction in the elderly: a matched analysis using propensity scores. J Clin Epidemiol. 2001;54(4):387–398. 11297888 10.1016/s0895-4356(00)00321-8

[42] Hernán MA, Robins JM. Causal Inference. Chapman & Hall; 2016.

[43] Causal inference: What if (the book). Miguel Hernan’s Faculty Website. October 19, 2012. Accessed October 16, 2024. https://www.hsph.harvard.edu/miguel-hernan/causal-inference-book/

[44] Danaei G, García Rodríguez LA, Cantero OF, Logan RW, Hernán MA. Electronic medical records can be used to emulate target trials of sustained treatment strategies. J Clin Epidemiol. 2018;96:12–22. 29203418 10.1016/j.jclinepi.2017.11.021 PMC5847447

[45] Bosworth M, Pawelek P, Ayoubkhani D. Prevalence of ongoing symptoms following coronavirus (COVID-19) infection in the UK - Office for National Statistics. February 2, 2023. Accessed October 11, 2024. https://www.ons.gov.uk/peoplepopulationandcommunity/healthandsocialcare/conditionsanddiseases/bulletins/prevalenceofongoingsymptomsfollowingcoronaviruscovid19infectionintheuk/2february2023

[46] Overview | COVID-19 rapid guideline: managing the long-term effects of COVID-19 | Guidance | NICE. Accessed August 26, 2024. https://www.nice.org.uk/guidance/ng188 33555768

[47] Hernán MA, Robins JM. Using Big Data to Emulate a Target Trial When a Randomized Trial Is Not Available. Am J Epidemiol. 2016;183(8):758–764. 26994063 10.1093/aje/kwv254 PMC4832051

[48] Hernán MA, Sauer BC, Hernández-Díaz S, Platt R, Shrier I. Specifying a target trial prevents immortal time bias and other self-inflicted injuries in observational analyses. J Clin Epidemiol. 2016;79:70–75. 27237061 10.1016/j.jclinepi.2016.04.014 PMC5124536

[49] CDC. Staying up to date with COVID-19 vaccines. COVID-19. August 12, 2024. Accessed August 14, 2024. https://www.cdc.gov/covid/vaccines/stay-up-to-date.html?CDC_AAref_Val=https://www.cdc.gov/coronavirus/2019-ncov/vaccines/stay-up-to-date.html

